# Enhancing the Therapeutic Efficacy of Daunorubicin and Mitoxantrone with Bavachinin, Candidone, and Tephrosin

**DOI:** 10.1155/2019/3291737

**Published:** 2019-11-07

**Authors:** Sina Darzi, Seyed Abbas Mirzaei, Fatemeh Elahian, Sadegh Shirian, Amir Peymani, Babak Rahmani, Shaghayegh Pishkhan Dibazar, Ehsan Aali

**Affiliations:** ^1^Department of Molecular Medicine, Faculty of Advanced Technologies, Shahrekord University of Medical Sciences, Shahrekord, Iran; ^2^Department of Pathology, Faculty of Veterinary Medicine, Shahrekord University, Shahrekord, Iran; ^3^Shiraz Molecular Research Center, Dr. Daneshbod Lab, Shiraz, Iran; ^4^Medical Microbiology Research Center, Qazvin University of Medical Sciences, Qazvin, Iran; ^5^Department of Molecular Medicine, Qazvin University of Medical Sciences, Qazvin, Iran; ^6^Department of Biotechnology, Qazvin University of Medical Sciences, Qazvin, Iran; ^7^Department of Pharmacology, Qazvin University of Medical Sciences, Qazvin, Iran

## Abstract

The capability of flavonoids in sensitizing cancer cells was demonstrated in numerous works to chemotherapy and converse multidrug resistance by modulating efflux pumps and apoptosis mechanisms. Three flavonoids, namely, bavachinin, tephrosin, and candidone, have been recently introduced to cancer treatment research presenting various activities, such as antibacterial, immunomodulatory, cell death, and anticancer. Less information exists regarding the therapeutic significance of these flavonoids in cancer treatment, especially in overcoming multidrug resistance (MDR). Here, we tempted to investigate the potency of these agents in reversing MDR by analyzing their effects as chemosensitizers on cell cytotoxicity, P-gp and ABCG2 protein expression levels, and their function on two multidrug-resistant cell lines, P-gp-overexpressing human gastric adenocarcinoma cell line (EPG85.257RDB) and ABCG2-overexpressing human epithelial breast cancer cell line (MCF7/MX). The inhibitory concentration of 10% (IC_10_) of bavachinin, tephrosin, and candidone in EPG85.257RDB cells was 1588.7 ± 202.2, 264.8 ± 86.15, and 1338.6 ± 114.11 nM, respectively. Moreover, these values in MCF7/MX cell were 2406.4 ± 257.63, 38.8 ± 4.28, and 27.9 ± 5.59 nM, respectively. Expression levels of ABCG2 and P-gp were not significantly downregulated by these flavonoids. Maximum levels of daunorubicin and mitoxantrone accumulations and minimum rates of drug efflux in both cell lines were detected 48 hrs posttreatment with tephrosin and bavachinin, respectively. Chemosensitization to mitoxantrone and daunorubicin treatments was, respectively, achieved in MCF7/MX and EPG85.257RDB cells in response to IC_10_ of bavachinin and tephrosin, independently. These effects did not follow time-dependent manner, and each flavonoid had its cell-dependent patterns. Overall, bavachinin, tephrosin, and candidone showed potency to sensitize MDR cells to daunorubicin and mitoxantrone and could be considered as attractive MDR modulators for cancer treatment. However, their action was time and cell specific.

## 1. Introduction

A major problem in cancer chemotherapy is drug resistance, not only to single, but to multiple drugs, which significantly compromises treatment outcomes. This phenotype is known as multidrug resistance (MDR), which is characterized by reduced intracellular drug accumulation leading to treatment failure. Variety of factors causes drug resistance; among them, overexpression of ATP-binding cassette (ABC) transporters is the most frequently occurring factor [1, 2]. So far, 49 members of human ABC transporter family have been discovered; among them, P-glycoprotein (P-gp, also referred to ABCB1 or MDR1) and ABCG2 (MXR or BCRP) which are the important members of ABC family attribute to MDR in cancer cells. These energy-dependent drug efflux transporters recognize and transport various chemotherapeutic agents out of the cell and consequently decrease intracellular drug levels and reduce their cytotoxic activity [3, 4]. Therefore, inhibiting and even reversing MDR have been an important goal for oncology researches [5, 6]. The most characterized and the first described ABC transporter is P-glycoprotein, a widely expressed protein with a broad spectrum of substrates and known to be responsible for the development of chemoresistance in cancer cells.

Nevertheless, ABC transporters are attracting interest as key players in carcinogenesis, and their activity often correlates with cancer progression and aggressiveness. As an example, P-gp is the best characterized multidrug resistance (MDR) protein, being the first human ABC transporters to be cloned. P-gp is known to transport a variety of hydrophobic drugs outside the cancer cells, thus conferring chemoresistance to numerous tumor types, such as gastric adenocarcinoma, breast cancer, pancreatic cancer, lung cancer, hepatocellular carcinoma, and neuroblastoma, leading to treatment failure and consequent tumor relapse.

The P-gp expression has been associated with tumor phenotype in colorectal cancer and soft tissue sarcomas, and its overexpression has also been linked with the progression of lymph node metastases. P-gp expression was also reported to be induced and elevated in chemoresistant breast and ovarian cancers. Furthermore, P-gp is involved in the resistance to apoptosis, which is one of the hallmarks of cancer cells. Inhibition of P-gp transporter results in cell cycle arrest and induction of apoptosis in leukemia and colon cancer, whereas its overexpression leads to cells being less responsive to apoptotic stimuli.

ABCG2 is known as breast cancer resistance protein (BCRP) and plays a role in multidrug resistance. Nevertheless, ABCG2 is mostly known for its role in multidrug resistance, being first described as breast cancer resistance protein or BCRP. ABCG2 is found to be overexpressed in numerous drug-resistant cancers including breast, ovarian, liver, lung, and melanoma, and it correlates with poor prognosis. Also, ABCG2 is found to be particularly overexpressed in a subpopulation of slow-cycling cancer stem-like cells with self-renewal capacity and high chemoresistance [7].

Various studies have recently demonstrated the capability of phytochemicals, such as flavonoids, to boost the cancer cells sensitivity to anticancer medications and inverse MDR through inhibiting ABC transporters [8]. Flavonoids, which are widely present in plants, may enhance the effectiveness of common cancer chemotherapy via preservative or synergistic impacts or by prompting chemosensitization in cancer cells. Moreover, cancer therapy-induced toxicity can be diminished by flavonoids while decreasing the threat of deleterious, unwanted complications of chemotherapeutic agents [9, 10]. These flavonoids exert their effects through various mechanisms, including inhibition of efflux pumps, cell death activation, and cell cycle arrest [11]. Lately, three flavonoids, namely, bavachinin [12], candidone [13], and tephrosin [14], have been introduced to cancer treatment research. Bavachinin is a flavonoid obtained from the seeds of *Psoralea corylifolia Linn* that displays various activities, including antiangiogenic, antitumor [15], antibacterial [16], antiallergic, and anti-inflammatory activities [17, 18]. Moreover, candidone, which is obtained from the stems and leaves of *Tephrosia candida* [19], exhibits antitumor [13] and antibacterial activities [20]. Tephrosin is isolated from *Amorpha fruticosa* (Leguminosae) and has inhibitory effects on human colorectal adenocarcinoma cell line [21], cancer cells invasion [22], and nuclear factor-kB activity [23].

**Scheme 1:**
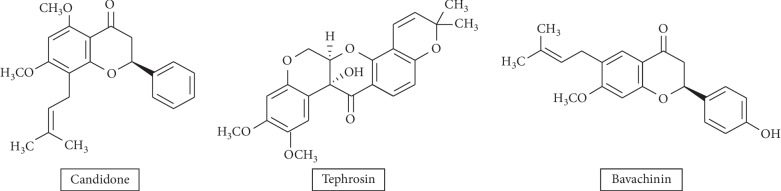


This study aimed at finding the effects of bavachinin, candidone, and tephrosin, as chemosensitizer, against ER-positive (estrogen receptor-positive) and PR-positive (progesterone receptor-positive) mitoxantrone (MX) resistant type of breast cancer cell line, MCF-7/MX, and the classical MDR cell line of gastric carcinoma resistant to daunorubicin, EPG 85.257 RDB cells. MCF-7/MX and EPG 85.257 RDB cells present the MDR phenotype, which is characterized by high expression levels of ABCG2 and P-gp transporters [24, 25]. Therefore, the impacts of these flavonoids were assessed in the current work on protein expression levels and activity of ABCG2 and P-gp transporters. The chemical structure of these flavonoids is given in Scheme 1.

## 2. Materials and Methods

### 2.1. Media and Chemicals

Trypsin, streptomycin and penicillin, RPMI-1640 medium (containing L-glutamine and high glucose), and fetal bovine serum (FBS) were prepared from Gibco (Grand Island, NY, USA). Chemotherapeutic agents (daunorubicin and mitoxantrone), thiazolyl blue tetrazolium bromide (MTT), Tween-20, phosphate-buffered saline tablet (PBS), bovine serum albumin (BSA), bavachinin, candidone, tephrosin, and MDR pump inhibitors (novobiocin sodium salt and verapamil hydrochloride) were bought from Sigma-Aldrich (Deisenhofen, Germany). P-gp and ABCG2 primary antibodies (mouse anti-P glycoprotein and anti-BCRP/ABCG2 monoclonal IgG, Abcam), anti-mouse secondary IgG1 of goat and FITC-conjugated IgG2a, and the suitable isotype controls were prepared from Abcam (Cambridge, USA). DMSO (dimethyl sulfoxide) was used to prepare the stocks of flavonoids and drugs, but to prepare different concentrations at the time of the experiment, a whole culture medium was used. DMSO, methanol, formaldehyde, and the other analytical-grade chemicals and solvents were obtained by Merck (Darmstadt, Germany).

### 2.2. Culturing the Cells and Cell Lines

P-gp-overexpressing human gastric adenocarcinoma cell line (EPG85.257RDB) and ABCG2-overexpressing epithelial breast cancer cell line of the human (MCF7/MX) were kindly offered by Professor Herman Lage (Medical University of Berlin, Charite Campus Mitte, Berlin, Deutschland). The cells were cultured in RPMI-1640 medium supplemented with 10% (v/v) FBS, antibiotics (100 *μ*/mL penicillin and 100 *μ*/mL streptomycin), and 2 mM L-glutamine. The cultured cells were incubated at the temperature of 37°C in a moistened atmosphere involving 5% CO_2_. Moreover, the culture media of the RDB and MX-resistant cell lines were supplemented with 100 nM mitoxantrone or 4.74 *μ*M daunorubicin, respectively [26, 27].

### 2.3. In Vitro Cytotoxicity Assessment

Cells were planted in 96-well plates at a density of 1,000 cells per well and were incubated at 37°C for 24 hrs. The cells were then treated with serial dilutions (0–15000 nM) of bavachinin, candidone, and tephrosin. In vitro cytotoxicity was measured following incubation for five days of the treated cells through MTT assay (at 570 nm) and via a BioTek microplate reader (ELX800™, USA). Inhibitory concentrations of 10% (IC_10_) are determined as the drug concentrations, decreasing the survival rates of the cells seeded in the wells to 10% in comparison with the control that was untreated cells. IC_10_ values were computed from the most optimal regression design of the proportion practicability versus the applied concentrations of each drug [27].

### 2.4. Flow Cytometry Analysis of Relative MDR Pump Levels

Resistant cancer cells were planted in 6-well tissue culture plates at a density of 5 × 10^5^ cells/well and were incubated at the temperature of 37°C. The culture media for MX- and RDB-resistant cell lines were supplemented with 100 nM mitoxantrone and 4.74 *μ*M daunorubicin, correspondingly [26, 27]. The cells were cultured in a medium without the drug for at least seven days before the experiments. Then, the cells were individually treated with IC_10_ of bavachinin, candidone, and tephrosin for 24, 48, and 72 hrs in 6-well plates. Afterward, the cells were gathered by trypsinization, fixed, and permeabilized by 10% (v/v) formaldehyde and 90% (v/v) ice-cold methanol for 10 min, respectively.

Through incubating the cells in PBS, including 10% (w/v) bovine serum albumin (BSA), nonexplicit binding sites were blocked for 1 hr at room temperature. The samples were then treated with each of P-gp (0.1 mg/ml) and ABCG2 (0.25 mg/ml, Abcam) monoclonal antibodies diluted (0.01 v/v) in PBS containing 2% BSA and 0.01% Tween-20 at 4°C. After that, the cells were incubated with the equivalent FITC-conjugated anti-mouse secondary immunoglobulin of goat (2 mg/ml) diluted (0.01 v/v) in PBS with 0.01% Tween-20 and 2% BSA within 20 min on ice in the dark. Rinsing with PBS at room temperature was performed two times within all the phases. Utilizing a BD FACSCalibur™ flow cytometer (BD Biosciences, USA) along with appropriate negative controls (secondary antibody, autofluorescence, and isotype controls), the protein levels of P-gp and ABCG2 were analyzed to diminish the nonprecise background signals. To perform the analysis, intact cells were separated from cellular clumps and cellular debris utilizing forward/side scatter gating. FITC-labeled proteins were excited using a regular argon laser at 488 nm, and via a 530/30 nm band-pass filter (FL1), the emission fluorescence strength was recorded. The obtained results were finally treated and analyzed via WinMDI (V.2.8) and FlowJo (version 7.6.1) in comparison with the corresponding untreated controls [27, 28].

### 2.5. Effects of Flavonoids on Kinetic of Chemotherapeutics Accumulation and Efflux

The impacts of flavonoids on the functionality of the MDR pump were quantified using flow cytometry. Concisely, the cells were planted in 6-well plates at a density of 5 × 10^5^/well and were individually treated using IC_10_ of bavachinin, candidone, and tephrosin for 24, 48, and 72 hrs. The cells were collected by trypsinization and equally allocated into two separate groups which were treated with fluorescent chemotherapeutic substrates, namely, daunorubicin and mitoxantrone (1 *μ*M for P-gp and 3 *μ*M for ABCG2 efflux pumps, respectively), individually or combined with explicit pump inhibitors, namely, verapamil (10 *μ*M for P-gp) and novobiocin (200 *μ*M for ABCG2). Then, the cells were incubated for 30 min at 37°C, after being gathered at 800 ×*g* and rinsed two times via ice-cold PBS. The suspended cells were correspondingly allocated into two parts; one half was incubated on ice in the dark and FACS analysis was immediately performed to investigate the accumulation kinetics, while the other half was treated using RPMI-1640 including 10% FBS supplemented with inhibitors (cells treated with inhibitor in the accumulation phases) or with no precise inhibitors (cells treated without inhibitor in the accumulation phases) for 1 hr at 37°C and was rinsed two times with ice-cold PBS. Then, FACS analyses were performed on these cells to study efflux kinetics.

To remove cell clumps and cellular debris, forward/side scatter gating was utilized. The cells were excited at 488 nm while recording the emission via a 585/42 nm band-pass filter (FL2, for daunorubicin) and a 670 nm long-pass filter (FL3, for mitoxantrone-treated cells). A total of 10^5^ happenings were recorded for each sample, and mathematical procedures were performed using equations (1) and (2). Modulator, MFI, specimen, and control are the pump inhibitors, mean fluorescent strength, flavonoid-treated cells, and untreated cells, respectively. All the experiments were performed in triplicate, and untreated cells were considered as the negative controls [27].

(1) $$\begin{array}{c} \Delta \text{efflux}=\left(\left(\underset{\left(\text{with modulator specimen}\right)}{\text{MFI}}-\underset{\left(\text{without modulator specimen}\right)}{\text{MFI}}\right)÷\;\left(\underset{\left(\text{with modulator control}\right)}{\text{MFI}}-\underset{\left(\text{without modulator control}\right)}{\text{MFI}}\right)\right)\times 100\%, \end{array}$$

(2) $$\begin{array}{c} \text{drug accumulation}=\left(\underset{\left(\text{without modulator specimen}\right)}{\text{MFI}}÷\underset{\left(\text{without modulator control}\right)}{\text{MFI}}\right)\times 100\%. \end{array}$$

### 2.6. Statistical Analysis

Statistical analyses were carried out by GraphPad Prism 6 software (GraphPad Software. San Diego, CA). The findings were provided as the mean ± standard deviation. One-way ANOVA with post hoc Dunnett test was utilized to compare the results within the groups. *P* values less than 0.05 were regarded as statistically significant. All the tests were carried out at least in triplicate.

## 3. Results

### 3.1. In Vitro Cytotoxicity of Flavonoids

Using MTT assay, the IC_10_ of bavachinin, candidone, and tephrosin on MDR-resistant lines was determined (MCF7/MX and EPG85.257RD). For this goal, after treatment with serial dilutions of the abovementioned flavonoids for five days, cytotoxicity values of each flavonoid were determined using a dose-response curve based on the obtained data. The IC_10_ values of the triplicate experiments were reported as the mean ± SE in Table 1. Tephrosin and candidone presented higher toxicity in MCF-7/MX than in EPG85.257RDB cells.

### 3.2. Relative Protein Quantification of MDR Pump

**Figure 1:**
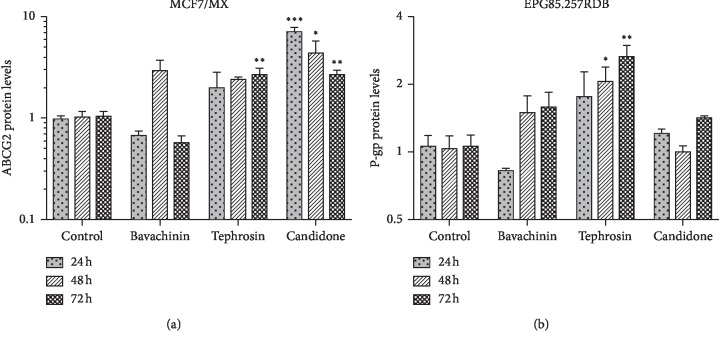
Relative quantification of MDR protein expression levels by flow cytometer. The protein levels of ABCG2 and P-gp in MCF7/MX and EPG85.257RDB were, respectively, quantified in the presence of bavachinin, candidone, and tephrosin using flow cytometry. Fold changes were calculated relative to the untreated control, and the data are presented as the mean of triplicate experiments ± SEM. The symbols ^*∗*^, ^*∗∗*^, and ^*∗∗∗*^ represent *P* values <0.05, <0.01, and <0.001, respectively. The *Y*-axis was plotted by log10 (a) and log2 (b).

The relative protein expression levels of P-gp and ABCG2 with and without IC_10_ of bavachinin, candidone, and tephrosin were assessed using flow cytometry.  Figure 1(a) illustrates the effect of bavachinin, candidone, and tephrosin on the protein expression level of ABCG2 in MCF7/MX cell line, and Figure 1(b) displays the impact of the abovementioned flavonoids on the expression level of P-gp protein in EPG85.257RDB cell line. According to Figure 1, the protein expression levels of ABCG2 and P-gp were not significantly downregulated by the studied flavonoids (*P* > 0.05). Candidone in MCF7/MX cells and tephrosin in both EPG85.257RDB and MCF7/MX cells significantly upregulated the expression levels of ABCG2 and P-gp proteins.

### 3.3. Effect of Flavonoids on MDR Transporter Activity

**Figure 2:**
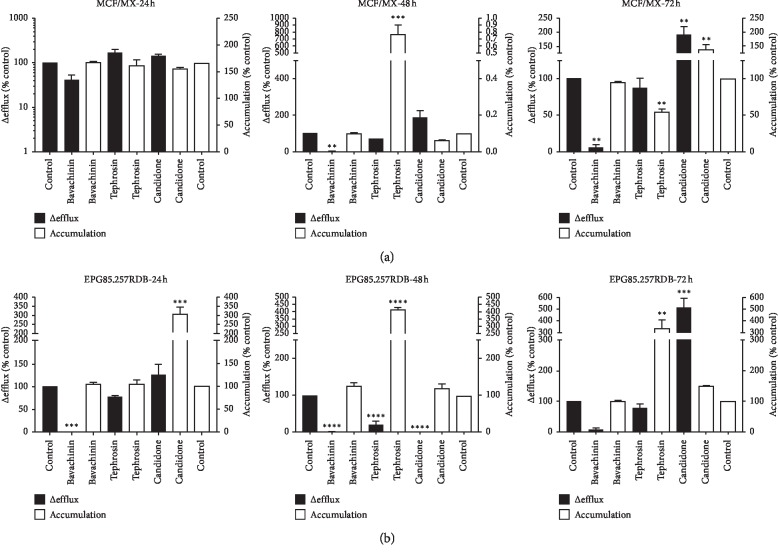
Effects of bavachinin, candidone, and tephrosin on the efflux of daunorubicin and mitoxantrone from MDR-resistant cells and their accumulation. MCF7/MX (a) and EPG85.257RDB (b) cells were treated with IC_10_ of flavonoids for 24, 48, and 72 hrs. The data are presented as the mean of triplicate experiments ± SEM. The symbols ^*∗*^, ^*∗∗*^, ^*∗∗∗*^, and ^*∗∗∗∗*^ represent *P* values <0.05, <0.01, <0.001, and <0.0001, respectively. The *Y*-axis was plotted by log10 (a, 24 h).

Following assessing the cytotoxicity and IC_10_ of the studied flavonoids, the modality of the effects of these flavonoids on mitoxantrone and daunorubicin accumulation in MCF7/MX (Figure 2(a)) and EPG85.257RDB cells was evaluated (Figure 2(b)). Efflux of mitoxantrone from MCF7/MX cells was considerably decreased by bavachinin in a time-dependent mode without any alteration in its accumulation. Treatment of MCF7/MX cells with tephrosin for 48 hrs resulted in the maximum accumulation of mitoxantrone. However, this maximum accumulated amount of mitoxantrone was significantly decreased after 72 hrs of tephrosin treatment. Efflux of mitoxantrone from MCF7/MX cells and its accumulation were significantly increased following 72 hrs of treatment with candidone.

Accumulation of daunorubicin in EPG85.257RDB cells was significantly enhanced during the first 24 hrs of exposure to candidone (*P* < 0.001). Daunorubicin efflux from EPG85.257RDB cells was significantly decreased by candidone after 48 hrs of treatment without any alteration in its accumulation level (*P* < 0.0001). Although 72 hrs of treatment of the cells with tephrosin resulted in an increase in the accumulated amount of daunorubicin (*P* < 0.01), the maximum accumulation level of daunorubicin in EPG85.257RDB cells was observed after 48 hrs of tephrosin exposure (*P* < 0.0001). An appropriate time-dependent manner in accumulation of daunorubicin in the cells could not be detected in this study. Interestingly, both bavachinin and tephrosin significantly reduced the activity of P-gp pump in EPG85.257RDB cell line. However, only a 48 hrs treatment with tephrosin could result in a decreased efflux and an increased accumulation of daunorubicin up to the maximum rate in EPG85.257RDB cells (*P* < 0.0001). Even though the treatment of the cells with bavachinin did not considerably alter the accumulation of daunorubicin and mitoxantrone in both the abovementioned cell lines, the minimum level of drug efflux was detected 48 hrs after bavachinin exposure.

## 4. Discussion

MDR transporters are involved in cross-resistance against structurally and functionally different anticancer agents, which results in chemotherapy failure. Daunorubicin and mitoxantrone are commonly used chemotherapeutic agents for the treatment of advanced human cancers. However, their long-term administration leads to drug resistance. Therefore, vast efforts have been made to search for new substances that effectively modulate drug resistance by targeting efflux pumps. There is ample evidence that flavonoids could protect us from cancer (chemopreventive) or enhance the anticancer effects of chemotherapy (chemosensitizers) or even fight against cancer [29]. The strategy of combining chemotherapeutic agents with flavonoids to reverse MDR is a promising alternative for reaching higher curability with lower toxicity. Three flavonoids, namely, bavachinin [12], candidone [13], and tephrosin [14], have been recently introduced to cancer treatment research. In this study, breast cancer (MCF7/MX) and gastric adenocarcinoma (EPG85.257RDB) resistant cell lines were applied, to investigate the effect of these flavonoids upon MDR. Overall, bavachinin, tephrosin, and candidone showed potency to sensitize MDR cells to daunorubicin and mitoxantrone. Moreover, their action is time and cell specific.

Despite the constant protein expression of ABCG2 and P-gp in bavachinin-treated cells, efflux of mitoxantrone from MCF7/MX cells was significantly reduced by this flavonoid (Figure 2(a)). However, this effect in daunorubicin was limited to 24 and 48 hrs posttreatment in EPG85.257RDB cells (Figure 2(b)). Recent studies have reported various activities and properties for bavachinin, including inhibition of nitric oxide production [30], pan PPARs (peroxisome proliferator-activated receptors) agonist [31], antiangiogenic, antitumor [15], antibacterial [16], antiallergic, and anti-inflammatory activities [17, 18]. Few studies have investigated the antitumor mechanism of bavachinin. Nepal et al. (2012) have conducted a study to investigate the angiogenesis effect of bavachinin both in vitro and in vivo. Their results indicated that bavachinin induced antiangiogenic and antitumor effects by targeting hypoxia-inducible factor-1a [15]. However, the potential of clinical application of bavachinin may be limited due to its low water solubility (<30 ng/mL), rapid clearance, toxicity, and subsequent liver injury [32, 33]. The toxicity of bavachinin is partly induced by the oxidative damage through p38/JNK MAPK (Jun N-terminal kinase and p38 mitogen-activated protein kinase) pathways [33] and potent inhibitory effects against human UDP-glucuronosyltransferase 1A1 [34]. Therefore, an alternative delivery method should be developed to achieve effective treatment [32].

The protein expression level of ABCG2 was significantly upregulated in the candidone-treated MCF7/MX cells (Figure 1(a)), which was followed by an increase in mitoxantrone efflux (Figure 2(a)). In EPG85.257RDB cells, although candidone decreased daunorubicin efflux after 48 hrs, regardless of the P-gp protein expression and daunorubicin accumulation, and increased efflux after 72 hrs (Figure 2(b)). Few studies that have investigated the effects of candidone have reported weak antitumor [13] and moderate antibacterial activities for this compound [20]. In contrast to candidone, tephrosin significantly increased P-gp protein expression level in EPG85.257RDB cells (Figure 1(b)), and interestingly, augmented daunorubicin accumulation and decreased efflux after 48 hrs treatment (Figure 2(b)). Tephrosin did not affect ABCG2 protein expression while it increased mitoxantrone accumulation after 48 hrs. However, the protein expression level of ABCG2 in MCF7/MX cells was relatively upregulated 72 hrs after tephrosin treatment (Figure 1(a)). Moreover, tephrosin treatment decreased mitoxantrone accumulation (Figure 2(a)). Tephrosin, as an anticancer agent [22], prevents cancer growth in human colorectal adenocarcinoma cells [21], inhibits cancer cells invasion, induces differentiation activity in leukemic cells [22], and inhibits the activity of nuclear factor-kB [23]. Anticancer activity of tephrosin may be implemented through the induction of epidermal growth factor receptor (EGFR) and ErbB2 internalization and degradation of colon cancer cells [35] as well as autophagic cell death [36]. Therefore, the combination of this agent with 2-deoxy-D-glucose (2-DG), a synthetic glucose analog acting as a glycolytic inhibitor, intensifies therapeutic efficacy of 2-DG by increasing the speed of ATP depletion and blunting autophagy [37].

In conclusion, based on the findings of the current work, ABCG2-overexpressing MCF7/MX cells and P-gp-overexpressing EPG85.257RDB cells potently became chemosensitizer to mitoxantrone and daunorubicin, after a 5-day treatment with bavachinin, tephrosin, and candidone flavonoids. Maximum daunorubicin and mitoxantrone accumulation levels and minimum levels of drug efflux in both cell lines were detected 48 hrs posttreatment with tephrosin and bavachinin, respectively. The effects of the studied flavonoids did not follow a time-dependent manner, and each flavonoid had its cell-dependent patterns. Also, there was no significant balance between the reduced efflux and increased accumulation responses in most of the treatment groups. These results indicated that bavachinin, candidone, and tephrosin could be considered as attractive candidates of MDR modulators for multidrug-resistant cancer treatment. However, further studies are required to find their toxicity levels, suitable delivery methods, and mechanism of actions.

Consequently, flavonoids are present in a variety of plant foods, and they have few side effects. On the other hand, their anticancer and cancer prevention effects have proven. With the results of this study, as well as future complementary studies, one of these flavonoids, or a combination of them, can be used as a MDR modulator, for enhancing the therapeutic efficacy of anticancer drugs.

However, in vivo studies and investigations into the effects of flavonoids on the human body, as main challenges, are still unclear.

## Figures and Tables

**Table 1:** IC_10_ values of bavachinin, candidone, and tephrosin for resistant cells.

Cell line	Bavachinin	Tephrosin	Candidone
EPG85.257RDB	1588.7 ± 202.2	264.8 ± 86.15	1338.6 ± 114.11
MCF7/MX	2406.4 ± 257.63	38.8 ± 4.28	27.9 ± 5.59

The values are expressed as flavonoids IC_10_ ± SE (nM).

## Data Availability

The reanalysed flow cytometry data used to support the finding of this study are included within the article and available from the first author upon request.
